# Acute Toxicity Comparison of Single-Walled Carbon Nanotubes in Various Freshwater Organisms

**DOI:** 10.1155/2015/323090

**Published:** 2015-01-14

**Authors:** Eun Kyung Sohn, Young Shin Chung, Seyed Ali Johari, Tae Gyu Kim, Jin Kwon Kim, Ji Hyun Lee, Yong Hwa Lee, Sung Wook Kang, Il Je Yu

**Affiliations:** ^1^Institute of Nanoproduct Safety Research, Hoseo University, Asan 336-795, Republic of Korea; ^2^Fisheries Department, Faculty of Natural Resources, University of Kurdistan, P.O. Box 416, Sanandaj, Kurdistan 66177-15175, Iran; ^3^Toxicity Evaluation Team, Korea Conformity Laboratories, Incheon 406-840, Republic of Korea

## Abstract

While the commercialization of single-walled carbon nanotubes (SWCNTs) is rapidly expanding, the environmental impact of this nanomaterial is not well understood. Therefore, the present study evaluates the acute aquatic toxicity of SWCNTs towards two freshwater microalgae (*Raphidocelis subcapitata* and *Chlorella vulgaris*), a microcrustacean (*Daphnia magna*), and a fish (*Oryzias latipes*) based on OECD test guidelines (201, 202, and 203). According to the results, the SWCNTs inhibited the growth of the algae *R. subcapitata* and *C. vulgaris* with a median effective concentration (EC_50_) of 29.99 and 30.96 mg/L, respectively, representing “acute category 3” in the Globally Harmonized System (GHS) of classification and labeling of chemicals. Meanwhile, the acute toxicity test using *O. latipes* and *D. magna* did not show any mortality/immobilizing effects up to a concentration of 100.00 mg/L SWCNTs, indicating no hazard category in the GHS classification. In conclusion, SWCNTs were found to induce acute ecotoxicity in freshwater microalgae, yet not in *D. magna* and medaka fish.

## 1. Introduction

Carbon nanotubes (CNTs) are the third most important engineered nanoparticles (ENP) listed in consumer product inventories [1]. The spatial structures of CNTs give them the unique properties (strength, hardness, thermal conductivity, microwave absorption capabilities, electrical properties, and catalytic properties) that are related to their many applications in such industrial and biomedical fields as nanocomposites, electronic devices, wastewater treatment, and drug delivery systems [2–4]. There are two types of manufactured CNTs: multiwalled carbon nanotubes (MWCNTs, 2~30 concentric cylinders with outer diameters 30~50 nm) and single-walled carbon nanotubes (SWCNTs, single-layered graphitic cylinders). On a worldwide basis, an estimated 33 tons of CNTs enter water bodies annually [5]. Plus, the presence of CNTs in aqueous environments has been predicted to range from 0.0005 to 0.0008 micrograms per liter [6]. Therefore, understanding the effects of CNTs on aquatic organisms has become particularly relevant.

The environmental risks of CNTs and methodological considerations for testing their toxicity have already been addressed in various reports and reviewed in recent publications [7, 8]. For example, well-dispersed MWCNTs in an aqueous system have been found to induce more significant developmental toxicity in zebrafish embryos than agglomerated MWCNTs [9]. Meanwhile, among four different carbon nanomaterials (SWCNT, MWCNT, C60, and carbon black), SWCNTs exhibited the most toxicity when exposing* D. magna* to a median lethal concentration of 2.425 mg/L [10]. Plus, the exposure of rainbow trout to SWCNTs was found to cause an increase in the total glutathione levels in the gills and liver and damage in the respiratory system [11]. Both natural organic material (NOM) coated CNTs and functionalized CNTs were also found to increase the toxicity in aquatic organisms [12, 13]. Freshwater algae (*Pseudokirchneriella subcapitata*) exposed to SWCNTs showed a decreased growth rate, with an inhibition concentration (IC_25_) of 1.04 mg/L during 72 h [14]. The growth of* C. vulgaris *was also inhibited by CNTs with EC_50_ values of 1.8 and 24 mg/L for well-dispersed and agglomerated suspensions, respectively [15].

Yet, since most previous aquatic toxicology experiments with specific SWCNTs were conducted using a single species at a time, the present study tested the acute toxicity of raw SWCNTs using four different freshwater species, including two freshwater microalgae (*Raphidocelis subcapitata* and* Chlorella vulgaris*), a microcrustacean (*Daphnia magna*), and a fish (*Oryzias latipes*). In addition, to investigate the role of a dispersant on the stability of raw SWCNTs in an aquatic system, five different dispersants were evaluated for their dispersion capability.

## 2. Materials and Methods

### 2.1. Materials

The SWCNTs were obtained from Hanwha Nanotech (Incheon, Korea), the first Korean manufacturer and supplier of CNTs and related products. The black powder was called ASA-100F (Lot number A-120229), and according to the scanning electron microscope (JEOL JSM-6700F SEM) information provided by the manufacturer, the SWCNTs had a tangled shape and were ~20 *μ*m in length and 1~1.2 nm in diameter. Plus, the purity was 30% based on the thermogravimetric analyzer (Q500, TA Instruments) information provided by the manufacturer. A TEM analysis of the dispersed SWCNTs was also performed at Inha University. The bovine serum albumin was purchased from Sigma, and the M4 and algae media were reconstructed based on OECD TG 202 and 203.

### 2.2. Metal Elementary Composition Method

The metal impurities in the SWCNTs were analyzed using an inductively coupled plasma mass spectrometer (ICP-MS, ELAN DRC II Axial Field Technology, PerkinElmer Sciex, USA). Briefly, 0.2 g samples were acidified with 2 g of hydrogen peroxide and 2 g of nitric acid in a heating block at 100°C. The samples were then acidified with 2 g of hydrofluoric acid and diluted with deionized water to make 20 mL. After syringe-filtering the acidified and diluted samples using PTFE 0.2 *μ*m, the SWCNTs were found to contain cobalt (Co) 5.339%, ferrous sulfate (FeSO_4_) 2.708%, nickel (Ni) 2.673%, and copper (Cu) 0.004%.

### 2.3. Dispersion Media and Stability

To select a good dispersant for the preparation of the SWCNT suspensions, stability tests were conducted using distilled water (D.W.) and five candidate dispersants: (1) gum arabic (GUM), (2) 1,2-dipalmitoyl-*sn*-glycero-3-phosphocholine (DPPC), (3) 1% Pluronic F-68 (PLURONIC), (4) 5% bovine serum albumin (BSA), and (5) lysophosphatidylcholine (LPC). The results of the stability tests conducted over a period of two weeks were then used to select the best dispersant.

### 2.4. Freshwater Microalgae Toxicity Test

The acute toxicity of the SWCNTs was tested using* Raphidocelis subcapitata* and* Chlorella vulgaris* in accordance with standard OECD guideline number 201 (freshwater alga and cyanobacteria, Growth Inhibition Test) [16]. The* Raphidocelis subcapitata* (ATCC 22662) was purchased from the American Type Culture Collection and tested prior to the study to determine its exponential growth in an inoculums culture. The* Chlorella vulgaris* (CCAP 211/11b) used in the toxicity tests was received from the National Institute of Environmental Research (NIER), Incheon, Korea, and originally purchased from the Culture Collection of Algae and Protozoa (CCAP).

All the algae tests were conducted using an initial cell density of 1.0 × 10^4^ cells/mL at 20 ± 1°C with continuous shaking at 100 rpm and under an illumination of 4,000~6,000 lux.

The SWCNTs were dispersed in distilled water containing 0.5% BSA. Each species of microalgae was exposed in triplicate to five different concentrations of dispersed SWCNTs (12.00 to 46.10 mg/L for* Raphidocelis subcapitata* and 15.00 to 42.84 mg/L for* Chlorella vulgaris*). Also, two different controls, including distilled water and the dispersant, were used for comparison. The algal biomass was determined every 24 hours for 72 hours based on cell counting using a hemocytometer under a microscope. The average specific growth rate was determined as the biomass increase after 72 hours using (1), where *μ*_*i*−*j*_ is the average specific growth rate from time *i* to time *j*, *t*_*i*_ is the initial time of exposure period, *t*_*j*_ is the final time of exposure, *C*_*i*_ is the biomass at time *i*, and *C*_*j*_ is the biomass at time *j*. Plus, the percentage inhibition of growth was calculated using (2), where %*I*_*r*_ is the percentage inhibition of the average specific growth rate, *μ*_*c*_ is the mean value of the average specific growth rate in the control group, and *μ*_*T*_ is the average specific growth rate after treatment. Consider

(1) $$\begin{array}{c} \mu_{i-j}=\frac{\left(\ln C_{j}-\ln C_{i}\right)}{t_{j}-t_{i}}, \end{array}$$

(2) $$\begin{array}{c} \%I_{r}=\left\{\frac{\left(\mu_{c}-\mu_{T}\right)}{\mu_{c}}\right\}\times 100. \end{array}$$

The median effective concentration (EC_50_) was estimated based on the average specific growth rate and percentage inhibition of average specific growth.

### 2.5. Freshwater Microcrustacean Toxicity Test

The acute toxicity of the SWCNTs was also tested using* Daphnia magna* in accordance with standard OCED guideline number 202 (*Daphnia* sp., Acute Immobilization Test) [17]. The* D. magna* were obtained from the GLP Center at Hoseo University and cultured in fully aerated M4 media at 21 ± 1°C under a 16 : 8 h light/dark photoperiod. The SWCNTs were dispersed in distilled water containing 0.24% BSA and the* Daphnia* exposed to six different concentrations, including 3.13, 6.25, 12.50, 25.00, 50.00, and 100.00 mg/L, as well as 2 controls of distilled water and the dispersant (0.24% BSA) for 48 hours under a static system. Five neonates (younger than 24 h old) were used for each concentration based on 4 replications.

The movement, intoxication symptoms, abnormal behavior, and mortality of the* Daphnia* were carefully checked at 24 and 48 hours after exposure. The* Daphnia* were recorded as immobile if no movement was observed within 15 s after gentle agitation of the test vessel. In addition, the live* Daphnia* were categorized in one of the following groups according to their swimming type: normal swimming (Nor), erratic swimming (ERR), mainly at the bottom (BOT), and mainly at the surface (SUR). Any visible uptake and adsorption of SWCNTs by the* Daphnia* were monitored and observed under a microscope (Olympus CX41).

### 2.6. Freshwater Fish Toxicity Test

The acute toxicity of the SWCNTs was also tested using Japanese medaka (*Oryzias latipes*) in accordance with standard OECD guideline number 203 (fish, acute toxicity test) [18]. The test organisms were obtained from the Gangnam Aquarium and maintained in dechlorinated tap water at 24 ± 1°C under a 16 : 8 h light/dark photoperiod. The SWCNTs were dispersed in distilled water containing 0.01% BSA and sonicated six times for 30 minutes in an ultrasonic bath sonicator.

Four concentrations of 100.00, 50.00, 25.00, and 12.50 mg/L were selected for the acute toxicity tests. The fish were exposed to the SWCNTs based on a static exposure regime. Seven healthy fish were directly transferred into each prepared concentration in triplicate tests. Control groups (7 fish in 3 replicates), plus an additional vehicle control group (0.01% BSA), were also included. Any abnormality or mortality was recorded at 24, 48, 72, and 96 hours after exposure.

### 2.7. Statistical Analysis

As appropriate, the IC/EC/LC and their associated 95% confidence intervals (95% CI) for the algae,* Daphnia*, and fish tests were calculated using the U.S. EPA Probit Analysis Program (version 1.5). If required, the statistical analyses were carried out using standard ANOVA techniques, followed by Tukey's significant difference test (SPSS ver. 17.0). Differences were regarded as statistically significant when *P* < 0.05.

## 3. Results

### 3.1. Dispersant Selection for SWCNTs

**Figure 1:**
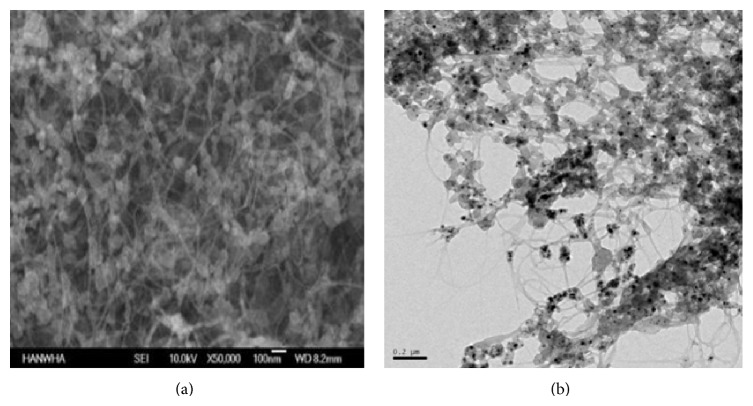
SEM image of undispersed SWCNTs ((a) ×50,000) and TEM image of dispersed SWCNTs ((b) ×100,000).

Figure 1 shows a SEM image of the raw SWCNTs and a TEM image of the dispersed SWCNTs in 0.5% BSA. Briefly, among the five different dispersants that were tested, DPPC, PLURONIC, LPC, and BSA accompanied with sonication produced a well-dispersed phase. However, in the case of DPPC and LPC, the SWDNTs were precipitated after 24 hours and one week, respectively, whereas 1% Pluronic and 0.5% BSA showed a proper suspension capacity for 2 weeks. Therefore, 0.5% BSA was finally selected as the dispersant for the hydrophobic SWCNTs and appropriate concentrations of BSA were individually selected for each organism.

### 3.2. Growth Inhibition Test of Microalgae

**Figure 2:**
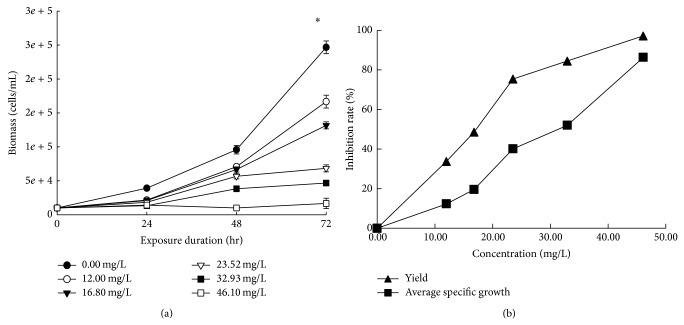
Growth inhibition of* Raphidocelis subcapitata* biomass caused by SWCNTs. Growth curves according to exposure duration (a) and percentage inhibition of average specific growth rate and yield according to concentration (b). ∗ Data represented mean ± SD.

**Figure 3:**
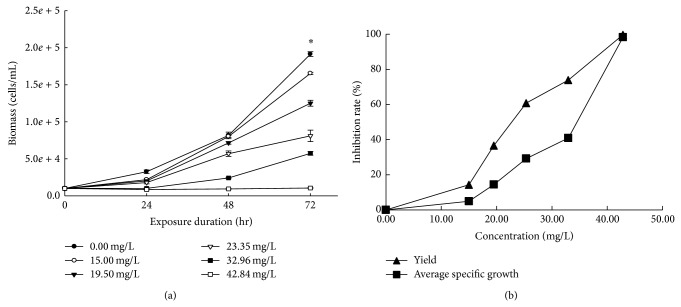
Growth inhibition of* Chlorella vulgaris* biomass caused by SWCNTs. Growth curves according to exposure duration (a) and percentage inhibition of average specific growth rate and yield according to concentration (b). ∗ Data represented mean ± SD.

The experimental conditions were properly controlled during the 72 hr incubation period. The temperature and fluorescent illumination in the shaking incubator were maintained at 24°C and 4,600~6,300 lux, respectively. The media pH measured before and after the incubation ranged from 7.49~7.63 to 7.34~7.71, respectively. The media turned green due to the algae, and turbidity of the media and agglomeration of SWCNTs were observed with all the treatments, except for the controls. The morphological observations of the algal cells are presented in Table 1, where flocculation, rupture, or atrophy was caused according to the concentration of SWCNT exposure. Plus, Figures 2 and 3 show the average specific growth and percentage inhibition of growth and yield of the algae during the 72 hr exposure to different concentrations of SWCNTs.

#### 3.2.1. Growth Inhibition Test of* Raphidocelis subcapitata*

After 72 hours of incubation, the biomass of* R. subcapitata* in the control and vehicle control increased about 17 and 21 times, respectively. However, when exposed to SWCNT concentrations of 12.00, 16.80, 23.52, 32.93, and 46.10 mg/L, the algal biomass increased 24.6, 16.7, 13.2, 6.9, 5, and 2 times, respectively. Thus, after 72 hours of SWCNT exposure, the increase in the biomass was lower than that for the controls and inversely dependent on the exposure concentration. Accordingly, the 72-hour median effective concentration (EC_50_) of SWCNTs based on the yield and average specific growth rate was calculated as 18.32 and 29.99 mg/L, respectively (Table 2).

#### 3.2.2. Growth Inhibition Test of* Chlorella vulgaris*

While the biomass of* C. vulgaris* in the control and vehicle control after 72-hour incubation showed proper growth of algae, the algal biomass exposed to SWCNT inversely increased depending on the exposure concentration ranging from 15.00 to 42.84 mg/L of SWCNT. The EC_50_ values of SWCNT based on the yield and average specific growth rate for* C. vulgaris* were 24.06 and 30.96 mg/L, respectively (Table 2).

### 3.3. Acute Immobilization Test of* Daphnia magna*

**Figure 4:**
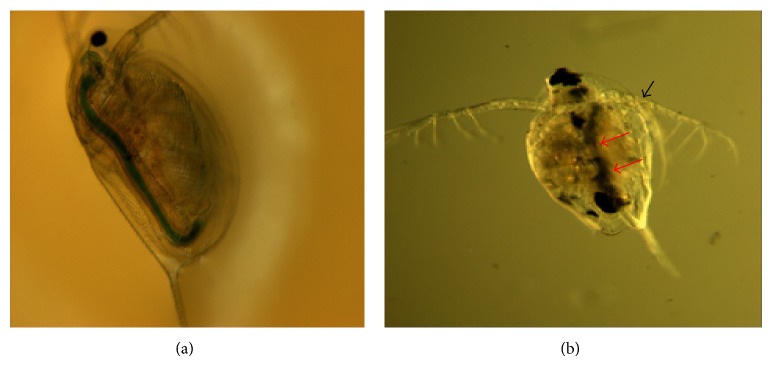
Microscopic images of* D. magna* after feeding with* Chlorella* (a) and after 48 hours of exposure to 25.00 mg/L SWCNTs (b). Arrows indicate the accumulation of SWCNTs in the gut (red) and on the antenna (black) of* Daphnia magna*.

The environmental conditions were properly controlled during the* Daphnia* toxicity experiments, where the temperature was in the range of 21.12~21.47°C, the pH was 7.23~7.46, and the hardness and alkalinity were 38 and 210 mg/L, respectively. No mortality/immobility was observed during the 48-hour exposure to the test concentrations of SWCNTs or in the controls; and only a few* Daphnia* were observed to swim abnormally or erratically. Thus, the 48 hr EC_50_ of SWCNTs for* D. magna* was estimated to be over 100.00 mg/L. Plus, microscopic observation revealed the accumulation of SWCNTs in the gut and on the antenna of the* Daphnia* (Figure 4).

### 3.4. Acute Toxicity Test of* Oryzias latipes*

No mortality was observed during the 96-hour exposure of the medaka fish to the test concentrations of SWCNTs or in the controls. Thus, the 96-hour LC_50_ was estimated to be over 100.00 mg/L. At the end of the 96-hour exposure, some fish excreted dark feces, which may be a sign of CNT ingestion by the animals. With the highest concentration of SWCNTs, the fish mostly swam near the water surface.

## 4. Discussion

This study attempted to understand one aspect of the aquatic toxicity of SWCNTs. The low solubility of hydrophobic CNTs in water media is one of the major limitations to studying their interaction with aquatic organisms. Therefore, BSA was carefully chosen for preparing the suspensions of SWCNTs and to maintain the stability of the dispersion phase for a long time. Distilled water and vehicle (BSA) controls were both used in all the toxicity tests, and the BSA was not found to induce any difference in the ecotoxicity from the distilled water.

The toxicity of the SWCNTs towards the freshwater algae* R. subcapitata* and* C. vulgaris* placed the raw SWCNTs in acute category 3 (10 mg/L < 72 or 96 hr EC_50_ for algae ≤100 mg/L) in the Globally Harmonized System of Classification and Labeling of Chemicals [19]. Several studies have already described the growth inhibition of freshwater algae by CNTs, which could be due to agglomeration, shading, photoactivity, and metal catalysts. For example, it has been reported that the growth inhibition was highly correlated with shading by the CNTs and agglomeration of the algal cells, yet not with photoactivity [15]. Also Long et al. [20] reported that the contribution of each of these factors changed depending on the size and concentration of MWCNTs. The major contributor at a lower concentration and smaller size was oxidative stress, whereas the contribution of shading and agglomeration increased when increasing the concentration. Another recent report showed that neither metal leaching nor shading, but photoactivity, contributed to the algal toxicity of commercial CNTs [21]. Wei et al. [22] also showed that functionalized MWCNTs interacted with the marine alga* Dunaliella tertiolecta* and induced oxidative stress on the cell surface. Notwithstanding, the mode of action regarding the growth inhibition of algae remains controversial. In the present study, shading may have been the major cause of growth inhibition in the freshwater algae system, as the tested SWCNTs had lower purity and photoactivity [21].

However, despite the good dispersion of SWCNTs on the one hand and low purity and high impurity of the SWCNTs on the other hand, the SWCNTs in this study did not cause any short-term acute toxicity against the* D. magna* and* O. latipes* up to a concentration of 100 mg/L. Yet, previous studies with invertebrates reported that the acute toxicity of CNTs increased with metal impurities, functionalization, and the presence of natural organic matter (NOM) [13, 23–26].

Although the current study found no signs of acute toxicity against the* Daphnia*, signs of SWCNT accumulation were observed in the animals' guts and antenna. Several other studies have also reported the accumulation of CNTs in the guts, antenna, and body surfaces of invertebrates [13, 23, 27]. Thus, the ingestion of CNTs by daphnids may lead to the subsequent transfer of CNTs to higher organisms in the food web.

Similarly, while this study found no signs of acute toxicity against the Japanese medaka, signs of SWCNT ingestion and excretion were observed; in addition, the fish swimming near the water surface may have been a sign of respiratory problems and limited oxygen uptake by the fish.

Severe acute toxicity has rarely been observed in studies with freshwater fish, yet toxic effects can appear in the case of long-term exposure. For example, the 10-day exposure of rainbow trout to SWCNTs was previously found to cause respiratory toxicity, including histopathological changes (oedema, altered mucocytes, and hyperplasia) in the gills [11]. Several studies have also reported that the exposure of zebrafish embryos to CNTs reduced survival and caused hatching delays and morphological abnormalities [28–30]. In addition, metal catalysts and functionalization, along with the short length of CNTs, have all been shown to have a negative effect on embryo hatching and the reproduction potential, as well as severe developmental toxicity [9, 12].

Thus, when taken together, the present results showed that SWCNTs could induce acute toxicity in freshwater algae (*Raphidocelis subcapitata* and* Chlorella vulgaris*), yet not in a freshwater microcrustacean (*Daphnia magna*) and freshwater fish (*Oryzias latipes*). Therefore, SWCNTs would seem to have different short-term effects on different species of aquatic organisms. Plus, more long-term chronic studies are needed to investigate how SWCNTs interact with aquatic organisms in relevant environmental concentrations.

## Figures and Tables

**Table 1:** Morphological observation of algal cells responding to SWCNT exposure.

*Raphidocelis subcapitata *		*Chlorella vulgaris *	
Concentration (mg/L)	Cell morphology	Concentration (mg/L)	Cell morphology
Control	Normal	Control	Normal
Vehicle control	Normal	Vehicle control	Normal
12.00	Flocculation	15.00	Flocculation
16.80	Flocculation	19.50	Flocculation
23.52	Flocculation, rupture, and atrophy	25.35	Flocculation, rupture, and atrophy
32.93	Flocculation, rupture, and atrophy	32.96	Flocculation, rupture, and atrophy
46.10	Flocculation, rupture, and atrophy	42.84	Flocculation, rupture, and atrophy

**Table 2:** Median effective concentration (EC_50_) based on yield and average specific growth rate of algae exposed to SWCNTs.

mg/L	*Raphidocelis subcapitata *		*Chlorella vulgaris *	
	Yield	Average specific growth rate	Yield	Average specific growth rate
EC_50_	18.32	29.99	24.06	30.96
95% confidence limits	13.38–22.88	28.15–32.04	21.08~27.06	26.74~37.04
